# Randomized, double-blinded, placebo-controlled pilot study: efficacy of faecal microbiota transplantation on chronic fatigue syndrome

**DOI:** 10.1186/s12967-023-04227-y

**Published:** 2023-07-29

**Authors:** Tapani Salonen, Elina Jokinen, Reetta Satokari, Perttu Lahtinen

**Affiliations:** 1 Department of Medicine, Tampere University Hospital, BOX 2000, FIN, 33521 Tampere, Finland; 2 Department of Gastroenterology, Tampere University Hospital, Tampere, Finland; 3 Human Microbiome Research Program, Faculty of Medicine, University of Helsinki, Helsinki, Finland; 4 Department of Gastroenterology, Päijät-Häme Central Hospital, Lahti, Finland

**Keywords:** Chronic fatigue syndrome, Health-related quality of life, Faecal microbiota transplantation

## Abstract

**Background:**

Chronic fatigue syndrome (CFS) is a disabling illness of unknown aetiology. Disruption of gut microbiota may play a role in several neurological disorders. In this study, the effect of faecal microbiota transplantation (FMT) on fatigue severity and health-related quality of life (HRQOL) in patients with CFS was evaluated.

**Methods:**

Randomized, placebo-controlled pilot trial. Patients and researchers were blinded to treatment assignment. 11 patients with CFS (10 female and 1 male, mean age 42.2 years and mean duration of CFS 6.3 years) were randomly assigned to receive either FMT from a universal donor (n = 5) or autologous FMT (n = 6) via colonoscopy. Patients’ HRQOL was assessed by using visual analog scale (VAS) and self-reporting questionnaires Modified Fatigue Impact Scale (MFIS), 15D and EQ-5D-3L. Patients’ HRQOL was evaluated at baseline, and 1 and 6 months after the FMT.

**Results:**

The baseline VAS scores in the FMT and placebo groups were 62.4 and 76.0 (p = 0.29). 1-month scores were 60.0 and 73.7 and 6-months scores 72.8 and 69.5, respectively. Total MFIS scores in the FMT and placebo groups were 59.6 and 61.0 at the baseline (p = 0.80), 53.5 and 62.0 at 1 month and 58.6 and 56.2 at 6 months. Compared to the baseline scores, differences at 1 and 6 months were statistically insignificant both in VAS and in MFIS. The 15D and EQ-5D-3L profiles did not change after the FMT or placebo. FMT-related adverse events were not reported.

**Conclusion:**

FMT was safe but did not relieve symptoms or improve the HRQOL of patients with CFS. Small number of study subjects limits the generalizability of these results.

*Trial Registration* ClinicalTrials.gov Identifier NCT04158427, https://register.clinicaltrials.gov, date of registration 08/08/2019.

## Background

Chronic fatigue syndrome (CFS) is a complex and disabling illness of unknown etiology and a heterogenous constellation of symptoms [1]. Notably, these include profound and persistent fatigue, post-exertional malaise, neurocognitive impairment, autonomic dysfunction, recurrent flu-like symptoms and gastrointestinal (GI) disturbances [2]. Health related quality of life (HRQOL) among CFS patients has been found to be markedly lower than in the general population [3]. The underlying pathomechanism of CFS has not been established, and no diagnostic tests exist. Instead, diagnosis relies on symptom-specific criteria to identify cases of CFS after all relevant differential diagnoses have been excluded [4]. Up to 75% of CFS cases report an infection-like episode preceding the onset of their illness [5].

The human microbiota is an extensive community comprising collectively over 10,000 different microbial species including bacteria, viruses and archaea inhabiting various anatomical regions, especially gastrointestinal tract [6]. Naturally occurring symbiotic or commensal microbiota have co-evolved with the human host and have shown beneficial host interactions including involvement in mediating physiological processes necessary for metabolic and immune function, digestion and nutrition [5, 7, 8]. Composition of microbiota is distinct for each person. Each body region itself contains substantial amount of diversity, particularly the gut. Multiple factors affect this composition. Both internal factors, such as the genetic background of the host, and external environmental and lifestyle factors can also greatly influence the microbiota [8]. Disruption of the integrity or equilibrium of these intricate microbial networks has been implicated in numerous pathological conditions or exacerbation of disease [8, 9].

There is increasing agreement that the disruption of the gut microbiota impacts brain development, neurological outcomes, and disorders, resulting in long-term behavioral changes [10]. Hence, gut dysbiosis is reported to be associated with several neurological disorders, including Alzheimer’s [11], Parkinson’s [12], Huntington’s disease [13], and multiple sclerosis [14]. Evidence for immunological aberrations in CFS suggests that the underlying pathomechanism may be due to enteric dysbiosis [10]. The proposed mechanism describes an alteration in the mucosal barrier function of the gut, which subsequently becomes hyperpermeable and allows increased translocation of commensal bacteria and their components into the bloodstream, potentially triggering a systemic chronic inflammatory immune response [15].

Faecal microbiota transplantation (FMT) is an emerging treatment method to renew dysbiotic gut microbiota. It has well established efficacy and acknowledged position in the treatment of recurrent Clostridioides difficile infection (rCDI) [16]. The good results in rCDI have encouraged the science community to explore FMT also in many non-communicable gut dysbiosis associated diseases including several neurological conditions [10].

CSF patients tend to have severely impaired HRQOL and they often remain without sufficient treatment options. Thus, we aimed to investigate the efficacy of reshaping the gut microbiota in CSF patients via FMT to improve HRQOL.

## Methods

The trial was approved by the ethical committee of Tampere University Hospital (identifier R18006) and written informed consent was obtained from all study participants.

Aim of the study: to evaluate the effect of FMT on HRQOL of patients with CFS.

### Patients

Altogether 128 patients with chronic fatigue were initially evaluated by Tampere University Hospital (TaUH) multidisciplinary specialist team between Jan 1st 2016 and May 1st 2019. TaUH is a tertiary-care hospital providing care for 540,000 inhabitants in the region. In all patients, a standardized protocol was applied to determine the cause of symptoms. Psychiatric assessment consisted of clinical evaluation by a psychiatrist. Patient-reported Beck’s Depression Inventory (BDI), Alcohol Use Disorders Identification Test (AUDIT) questionnaires and neuropsychological test were utilized. A neurologist ruled out neurological diseases based on the patient’s history and clinical condition. Nocturnal polysomnography was conducted to rule out sleep-related disorders. A specialist in internal medicine clarified patient’s medical history and a medical examination was conducted. Pre-determined laboratory tests were acquired to rule out diseases and medical conditions, which could cause fatigue (Table 1), and additional tests were obtained on the clinician’s decision.

**Table 1:** List of laboratory tests included in the evaluation of fatigue patients

Complete blood count
Erythrocyte sedimentation rate
Sodium, potassium
Creatinine
Thyroid-stimulating hormone, thyroxine
Cortisol
Testosterone (males)
Ferritin, transferrin saturation
Calcium
Vitamin B-12, Vitamin D
Blood fasting glucose, HbA1c
Alanine transaminase (ALT), alkaline phosphatase (ALP)
Antinuclear antibodies (ANA)
Creatine kinase (CK)
Ammonium ion
Blood gases
Epstein-Barr virus antibody test
Cytomegalovirus antibody test
Borrelia antibody test
HI-virus antigen and antibody test
Urinalysis
Electrocardigram (ECG)

In diagnosing CFS, the Institute of Medicine (IOM) criteria were applied [17]. The three required main symptoms were (1) a substantial reduction or impairment in the ability to engage in pre-illness levels of activity; (2) post-exertional malaise and (3) unrefreshing sleep. Moreover, at least one of two additional manifestations (cognitive impairment and orthostatic intolerance) was necessitated.

Of 128 patients, 29 patients were diagnosed with exclusively CFS and no other fatigue causing disease or condition and they were asked to enroll into the study. 16 patients refused to participate. The main reason was intense fatigue and inability to conduct the FMT procedure.13 patients agreed and provided signed written informed consent after being fully informed about the study protocol. Two patients withdrew their informed consent before randomization; one patient due to pregnancy and in one patient fatigue was too severe to accomplish the FMT. Eventually, 11 participants were enrolled into the study.

### Intervention

The 11 participants were randomly assigned in a double-blind fashion in a 1:1 ratio to receive either FMT or placebo. Randomization and preparation of transplants was performed by a study nurse. Patients in the FMT group received faecal transplantation from an exclusively tested universal donor [18]. Transplants were stored in a stool bank at – 80 °C and thawed immediately before colonoscopy [19]. The placebo group received a freshly prepared, autologous transplantation, i.e. a transplant prepared from patients own faeces. All patients were treated via a single colonoscopy with 30 g of faecal material administered into the caecum. To ensure blinding, both groups provided their stool for the preparation of placebo, and if the patient was randomized to the FMT group, the stool sample was discarded. Bowel preparation was performed using polyethylene glycol solution. No pre-treatment with antibiotics was given. The patients were not given any restrictions regarding nutrition or medications during the follow-up.

### Outcome

The prospectively defined main outcome measure was the change in fatigue scores assessed using visual analog scale and several validated self-reporting questionnaires. Patients’ HRQOL was evaluated at baseline, and 1 and 6 months after FMT.

Visual Analogue Scale (VAS): VAS measures fatigue intensity and consists of a 100 mm line, with two end points representing 0 (no fatigue) and 100 (extreme fatigue).

Modified Fatigue Impact Scale (MFIS): This instrument provides an assessment of the effects of fatigue in terms of physical, cognitive, and psychosocial functioning consisting of 21 items. Individual scores are generated by calculating the sum of specific sets of items.

15D: the 15D is a 15-dimensional instrument for measuring HRQOL among adult subjects. In each dimension, the 15D has five severity levels (1 = no problems, 5 = extreme problems/unable).

EQ-5D-3L: EQ-5D-3L consists of a descriptive system, which contains five items that each represent a health dimension, including mobility, self-care, usual activities, pain/discomfort, and anxiety/depression. In each dimension, the EQ-5D-3L has three severity levels (no, some, extreme problems/unable).

### Statistical analysis

Data are expressed as mean ± standard deviation (SD) unless otherwise stated. Patients’ characteristics were compared using the t-test for continuous variables.

## Results

Of 11 patients, 10 were female. Patients’ mean age was 42.2 (± 6.7) years and the mean duration of CFS symptoms at randomization was 6.3 (± 2.5) years. After randomization into FMT or placebo, patients’ age or duration of CFS did not differ statistically significantly (Table 2). In all study subjects, colonoscopy was conducted without complications and no FMT-related adverse events were reported.

**Table 2:** Patient characteristics

	FMT group (n = 5)	Placebo group (n = 6)	p value
Female	80%	100%	
Mean age at randomization (years) (range)	42,0 (32–49)	42,4 (29–52)	0,92
Duration on CFS (years) (range)	5,8 (5,0–6,7)	6,6 (3,0–11,3)	0,62

The average baseline VAS scores in the FMT and placebo groups were 62.4 (± 22.4) and 76.0 (± 3.6) (p = 0.29), respectively. Compared to the baseline scores, statistically significant changes at 1 or 6 months after the FMT/placebo were not found (Table 3.)

**Table 3:** VAS scores in the FMT and placebo groups

	Baseline	1 Month after the FMT	6 Months after the FMT	p value (Baseline vs. 1 Month)	p value (Baseline vs. 6 Months)
FMT group (n = 5)	62.4 (22.4)	60.0 (21.8)	72.8 (13.6)	0.88	0.45
Placebo group (n = 6)	76.0 (3.6)	73.7 (4.9)	69.5 (9.7)	0.41	0.20

Visual Analogue Scale (VAS) scores (mean) (± SD) in the FMT and the placebo groups. 0 = no fatigue, 100 = extreme fatigue. The difference in baseline scores between the groups was statistically insignificant (p = 0.29)

The MFIS scores are shown in the Table 4. There were not statistically significant differences in physical, cognitive, psychosocial or total scores between the groups. Neither the subscale scores nor the total scores changed after the FMT/placebo procedure.

**Table 4:** MFIS scores in the FMT and placebo groups

	Baseline	1 Month after the FMT	6 Months after the FMT	p value (Baseline vs. 1 Month)	p value (Baseline vs. 6 Months)
FMT group (n = 5)					
Physical subscale	29.4 (2,4)	26.2 (2.8)	27,4 (4,6)	0.08	0.42
Cognitive subscale	24.8 (7.5)	23.0 (8.0)	25.4 (8.9)	0.74	0.91
Psychosocial subscale	5.4 (1.1)	5.5 (1.3)	5.8 (1,5)	0.91	0.64
Total	59.6 (9.2)	53.5 (9.9)	58.6 (11.9)	0.45	0.79
Placebo group (n = 6)					
Physical subscale	30.0 (6,2)	29.5 (4.8)	25.7 (7.9)	0.88	0.32
Cognitive subscale	24.8 (4.5)	25.8 (8.2)	25.0 (11,1)	0.80	0.97
Psychosocial subscale	6.2 (1.0)	6.7 (1.2)	5.5 (2.3)	0.45	0.54
Total	61.0 (8.3)	62.0 (9.8)	56.2 (20.3)	0.85	0.61

Modified Fatigue Impact Scale (MFIS) scores (mean) (± SD) in the FMT and the placebo groups. Differences in the baseline scores between the groups were statistically insignificant (p values: Physical subscale 0.83; Cognitive subscale 0.99; Psychosocial subscale 0.27 and Total 0.80)

**Fig. 1:**
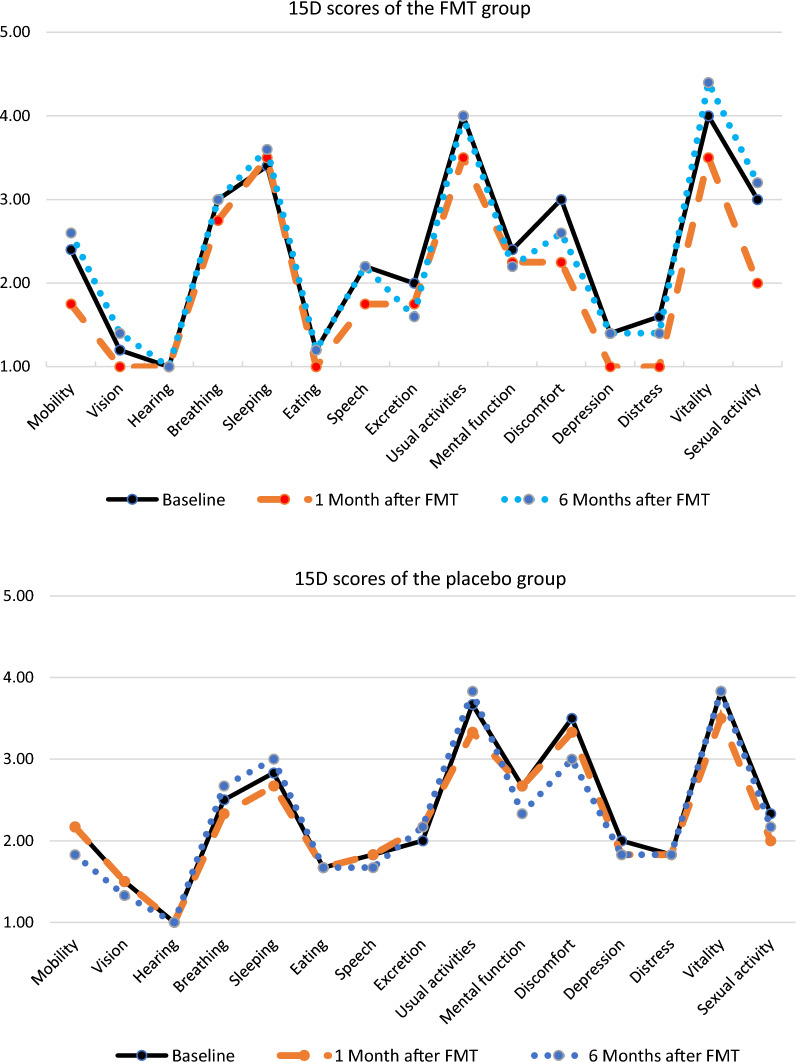
15D-scores in FMT and placebo groups. Severity levels in each dimension: 1 = no problems, 5 = extreme problems/unable. Compared to baseline scores, differences in 1 and 6 months scores are statistically non-significant

The 15D-profiles in the FMT and placebo groups are shown in the Fig. 1. In both groups, the highest scores reflecting severe reductions in HRQOL were found in dimensions assessing breathing, sleeping, usual activities, discomfort and vitality. After the FMT/placebo procedure, the profiles remained unchanged.

Table 5 shows EQ-5D-3L scores in the individual patients. There is some variety in the severity of symptoms between the subjects but there were no statistically significant changes in any dimension of single patient’s symptoms after the FMT/placebo. In the patient 3, 1 month data were not provided.

**Table 5:** EQ-5D-3L scores in the FMT and placebo groups

	Mobility	Self-care	Usual activities	Pain/discomfort	Anxiety/depression
FMT group					
Patient 1	1 1 1	1 1 1	2 2 2	2 1 2	1 1 1
Patient 2	2 2 2	2 2 2	3 3 3	2 1 2	1 1 1
Patient 3	3–3	2–2	3–3	3–3	2–1
Patient 4	2 2 2	2 2 2	3 3 3	2 2 1	1 1 1
Patient 5	2 2 2	1 1 1	3 2 3	2 2 2	1 1 1
Placebo group					
Patient 6	2 2 2	2 2 2	2 2 2	3 2 3	2 2 2
Patient 7	1 1 1	1 1 1	2 2 2	2 2 2	1 1 1
Patient 8	2 2 1	2 1 1	3 3 2	3 3 2	2 2 2
Patient 9	2 2 2	2 2 1	2 3 2	2 2 3	1 1 1
Patient 10	2 2 2	1 1 1	2 3 2	2 2 3	2 2 2
Patient 11	3 3 3	2 1 1	3 3 3	2 3 2	1 2 2

EQ-5D-3L profiles in individual patients. In each dimension, the first digit represents the severity of symptoms at baseline, the second and the third digits describe severity at 1 month and at 6 months after the FMT, respectively. 1 = no problems; 2 = some problems; 3 = extreme problems/unable. 1 month data of patient 3 missing

## Discussion

To our knowledge, this is the first randomized study to evaluate the effect of FMT on the HRQOL of patients with CFS. In this study, the IOM criteria were utilized to diagnose CFS. Several other case definitions have emerged. In 1994, Fukuda et al. introduced recommendations for the clinical evaluation of fatigued persons with key symptoms including post-exertional malaise, neurocognitive deficits and unrefreshing sleep [20]. The Clinical Canadian Consensus criteria (CCC) in 2003 [21] required seven symptoms selecting a smaller group of patients than the definition by Fukuda et al. [22]. Compared to Fukuda et al. and CCC clinical case definitions, IOM criteria bring in a larger group of patients, even though core symptoms are identified [23]. In this study, however, patients with severe fatigue were systematically evaluated by multidisciplinary specialist team. Strict criteria were applied and of 128 patients with fatigue, only 29 were diagnosed with CFS. In these patients, other fatigue causing diseases and conditions had been excluded, and they comprised a homogenous group without significant comorbidities.

There is no objective test to measure fatigue or HRQOL, but by utilizing a patient-reported outcome measure, professionals are able to gather insights with direct relevance to the patient questioned. Several different methods are applied to assess quality of life and there is no consensus on the best technique for determining patient well-being. In certain patient populations, substantial variability among commonly utilized questionnaires has been reported and discrepancies have been particularly apparent as data have been evaluated at the individual level. To avoid bias, we decided to apply an array of HRQOL measures [24].

FMT is commonly administered by three modalities: colonoscopy, oral capsules and gastroscope or nasojejunal probe [25]. Oral capsule is well tolerated and perhaps the most acceptable way by participants, while colonoscopy requires bowel lavage and is time consuming. In a recent meta-analysis, FMT delivered via colonoscopy was superior compared to oral capsules in patients with irritable bowel syndrome [26]. In this study, colonoscopy was applied to maximize the likelihood of successful FMT. However, a remarkable proportion of CFS patients refused to participate in the study while they considered the colonoscopy procedure being too burdensome for them.

In previous studies, HRQOL of CFS patient has been remarkably lower compared to the general population. In this study, 10 patients out of 11 reported their baseline VAS fatigue score over 50 (scale 0–100). The average total MFIS score in the FMT and placebo groups were 59.6 and 61.0, respectively. Besharat and colleagues reported mean MFIS score 46.7 in CFS patients compared to 25.4 in healthy controls [27]. Compared to patients with relapsing–remitting multiple sclerosis, the total MFIS score, and particularly physical and cognitive subscale scores were higher in our patients [28]. Prak and colleagues recently reported average total MFIS score 35.1 in patients with primary Sjögren’s syndrome [29].

The 15D is a standardized measure of HRQOL that can be used both as a profile and single index score measure representing overall HRQOL. It generically assesses health status in terms of 15 dimensions not specific to any patient group or disease. The index score (range 0–1) is calculated by using a set of population-based utility weights [30]. Similarly, the EQ-5D-3L is a widely used, simple and generic questionnaire for use in clinical trials. The descriptive system evaluates five dimensions of health with three levels of severity providing altogether 243 potential health states. Like the 15D-profiles, each EQ-5D-3L health state can further be converted into a single numerical value ranging from 0 to 1. These profile-based index scores are generally used in the economic health studies with large number of patients. In this report, the profiles are preferred instead of index scores to provide a comprehensive perspective of patients’ symptoms.

Our main hypothesis was that gut dysbiosis might explain the pathomechanism of CFS and that gut microbiota intervention with FMT could relieve CFS-related symptoms. We exclusively selected the CFS patients from a large group of patients with fatigue caused by other reasons. The study subjects were randomly allocated in a double-blind fashion to the FMT or the placebo groups. Colonoscopy was applied to administer the FMT. In a previous study using the same FMT procedure without prior antibiotic pre-conditioning, one dose of FMT was found to alter the microbiota of irritable bowel syndrome patients to resemble that of the donor [31].

During the six-month follow up, the HRQOL was assessed twice, and four different self-reported questionnaires were utilized. In this study, we could not find improvement in the HRQOL scores after the single FMT from an universal donor. There were no statistically significant differences in the characteristics or the baseline HRQOL scores between the FMT and the placebo groups. Compared to the baseline VAS and MFIS scores, the differences in 1- and 6-months scores were statistically non-significant in both the FMT and the placebo groups. There was a slight, but statistically non-significant improvement in the 1-month MFIS physical subscale scores. However, such tendency was not present in VAS scores. The individual patient’s EQ-5D-3L profiles remained practically unaltered throughout the study. The 15D profiles in the FMT and the placebo groups did not change during the follow-up. The limited number of study subjects inhibits the generalizability of these results. There was only one male subject in the study. To exclude gender-based bias, we re-analyzed the data after having the male subject removed from the analysis. The results did not change: no statistically significant difference was found between the FMT and the placebo groups. This pilot study did not confirm nor exclude the possibility that FMT could have some effect on the HRQOL of patients with CFS. The pathomechanism of CFS remains unknown and gut microbiota may affect the pathophysiology of several neurological disorders. Further research and larger trials including also evaluation of changes in bacterial composition are needed to discover the underlying mechanisms of CFS.

## Conclusion

In this pilot study, FMT was safe, but did not relieve symptoms or improve the HRQOL of patients with CFS. Small number of study subjects limits the generalizability of these results and further research is needed to assess the potential of FMT in CSF patients.

## Data Availability

The datasets used and analyzed during the current study are available from the corresponding author on reasonable request.

## Abbreviations

AUDIT: Alcohol use disorders identification test
BDI: Beck’s depression inventory
CCC: Clinical Canadian consensus criteria
CFS: Chronic fatigue syndrome
FMT: Faecal microbiota transplantation
HRQOL: Health-related Quality of Life (HRQOL)
IOM: Institute of medicine
MFIS: Modified fatigue impact scale
rCDI: Recurrent Clostridioides difficile infection
SD: Standard deviation (SD)
TAUH: Tampere university hospital
VAS: Visual analog scale
